# Vsx2 in the zebrafish retina: restricted lineages through derepression

**DOI:** 10.1186/1749-8104-4-14

**Published:** 2009-04-03

**Authors:** Marta Vitorino, Patricia R Jusuf, Daniel Maurus, Yukiko Kimura, Shin-ichi Higashijima, William A Harris

**Affiliations:** 1 Department of Physiology, Development and Neuroscience, University of Cambridge, Downing Street, Cambridge, CB2 3DY, UK; 2 National Institutes of Natural Sciences, Okazaki Institute for Integrative Bioscience, Okazaki, Japan

## Abstract

**Background:**

The neurons in the vertebrate retina arise from multipotent retinal progenitor cells (RPCs). It is not clear, however, which progenitors are multipotent or why they are multipotent.

**Results:**

In this study we show that the homeodomain transcription factor Vsx2 is initially expressed throughout the retinal epithelium, but later it is downregulated in all but a minor population of bipolar cells and all Müller glia. The Vsx2-negative daughters of Vsx2-positive RPCs divide and give rise to all other cell types in the retina. Vsx2 is a repressor whose targets include transcription factors such as Vsx1, which is expressed in the progenitors of distinct non-Vsx2 bipolars, and the basic helix-loop-helix transcription factor Ath5, which restricts the fate of progenitors to retinal ganglion cells, horizontal cells, amacrine cells and photoreceptors fates. Foxn4, expressed in the progenitors of amacrine and horizontal cells, is also negatively regulated by Vsx2.

**Conclusion:**

Our data thus suggest Vsx2-positive RPCs are fully multipotent retinal progenitors and that when Vsx2 is downregulated, Vsx2-negative progenitors escape Vsx2 repression and so are able to express factors that restrict lineage potential.

## Background

During vertebrate retinal neurogenesis, multipotent retinal progenitor cells (RPCs) either become restricted in their fate choice and proliferation potential or give rise to daughters that do so. These progenitors with restricted competence proliferate to a limited extent and then differentiate into six classes of neurons and one type of glia in a fairly conserved histogenetic order [1]. Transcription factors, particularly of the Homeobox and basic helix-loop-helix (bHLH) families then regulate the generation of specific cell types [2,3].

Factors that influence the multipotent state of retinal progenitors should be expressed in all early retinal progenitors. One such factor is the well-known transcription factor Pax6, which is critical for early eye formation [4]. The conditional inactivation of this gene later in development restricts the competence of retinal progenitors such that they differentiate only as amacrine cells [5], suggesting that *Pax6 *is part of a repertoire of genes that enables the multipotent retinal progenitor state. Another transcription factor expressed in all early progenitors is the homeodomain containing repressor Vsx2 (also called Chx10). Vsx2 is expressed early in all progenitors but later becomes restricted to bipolar neurons [6,7]. In the mouse, homozygous-null mutations in *vsx2 *cause microphthalmia [8,9], but in these small eyes bipolar cell fate is severely impaired. The effects of Vsx2 on proliferation and bipolar cell fate are separable, as cell number, but not bipolar cell fate, can be rescued by knocking out the cell cycle inhibitor p27kip1 [10]. The role of Vsx2 in bipolar cell fate is thought to be due to the fact that it represses genes involved in other fates [11-13], a concept consistent with the following three findings. First, postnatal knock down of Vsx2 leads to an increase of rods at the expense of bipolar cells, while misexpression causes the reverse phenotype: more bipolars, less rods [3,14-18]. Second, when Vsx2 is converted to an activator, bipolar cell fate is impaired [16], suggesting that its repressive activity is required for bipolar cell fate. Third, Dorval *et al*. [12] used yeast one hybrid, gel shift, and ChIP assays to show that Vsx2 interacts directly with the rhodopsin and other photoreceptor specific genes, including those encoding PCE-1 (rod arrestin/S-antigen) and the (red/green) cone opsin.

More recently, Vsx2 has also been shown to repress *vsx1*, which curiously belongs to the same paired-like class of homeobox genes and is also involved in bipolar cell differentiation [19-21]. Vsx2 binds to the *vsx1 *promoter and can repress the activity of a luciferase reporter under the control of this promoter [20].

Zebrafish Vsx2 shares 99% homology in the homeodomain and 100% in the CVC domain with mouse/human Vsx2 (Chx10) [19,21-24], regions described to be essential for its DNA binding activity [11]. In zebrafish, Vsx2 is strongly expressed in proliferating cells throughout the retinal neuroepithelium and is upregulated at later stages (62 hours post-fertilization (hpf)) in the outer part of the inner nuclear layer (INL), where bipolar cells are normally localized [23]. Treatment of zebrafish embryos with antisense oligonucleotides to *vsx2 *RNA showed a concentration-dependent reduction in the size of the eyes, resembling mouse and human microphthalmia in *vsx2 *mutants [25]. Therefore, as in mouse, Vsx2 is likely to be involved in proliferation. We were interested in whether Vsx2 also has a role in the determination of bipolar cell fate in zebrafish.

We use transgenic zebrafish, where fluorescent proteins, expressed under the control of *vsx2 *regulatory regions, allow us to follow the fate of Vsx2-expressing progenitors *in vivo*. We show that Vsx2 is present in all early RPCs, but turns off in most cells as retinogenesis proceeds, remaining on only in cells that become Müller glia or a specific subtype of bipolar cells. The daughter cells that lose Vsx2 expression give rise to lineages for other retinal cell types. For example, we demonstrate that Vsx2 represses *ath5*, which, like Vsx1, is expressed only in cells that turn off Vsx2. This enables the Ath5 lineage, which includes retinal ganglion cells (RGCs), amacrine cells, horizontal cells and photoreceptors to proceed. Combined with previous findings and other data in this paper, we suggest that early Vsx2-positive cells are the multipotent RPCs of the retina. This multipotent state becomes more restricted as Vsx2 is selectively repressed, permitting the generation of a variety of lineage restricted RPCs.

## Results

### Vsx2:GFP is expressed first in all RPCs and then later in bipolar and Müller cells in the developing zebrafish retina

**Figure 1:**
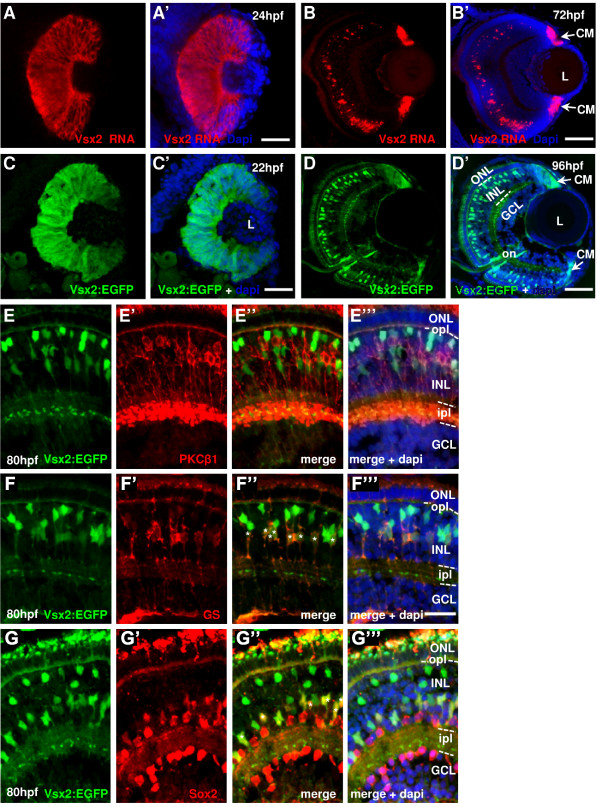
**Vsx2 expression in the zebrafish retina**. **(A-G) **Cryosections through the central retina of zebrafish embryos counterstained with DAPI (blue). Developmental stages are indicated by hours post-fertilization (hpf) at 28.5°C. (A, B) *vsx2 in situ *hybridization at 24 hpf (A) and 72 hpf (B). (C, D) Tg(*vsx2:GFP*) shows green fluorescent protein (GFP) expression in retinal progenitor cells (C) and in cells of the outer part of the INL (D). Arrows indicate *vsx2 *RNA (B) and Vsx2:GFP (D) expression in the ciliary margin (CM). (E-G) Tg(*vsx2:GFP*) expressing retinas labelled with the bipolar cell marker protein kinase C (PKC) at 80 hpf (E) and the two Müller cell markers glutamine synthetase (GS) (F) and Sox2 (G). No Vsx2:GFP-positive cells co-localise with the bipolar marker at 80 hpf (E). Müller cells expressing GS and Sox2 are also Vsx2:GFP-positive ((F", G") asterisks). GCL, ganglion cell layer; INL, inner nuclear layer; ipl, inner plexiform layer; L, lens; ONL, outer nuclear layer; opl, outer plexiform layer. Scale bars: (A-D) 100 μm; (E-G) 40 μm.

**Figure 2:**
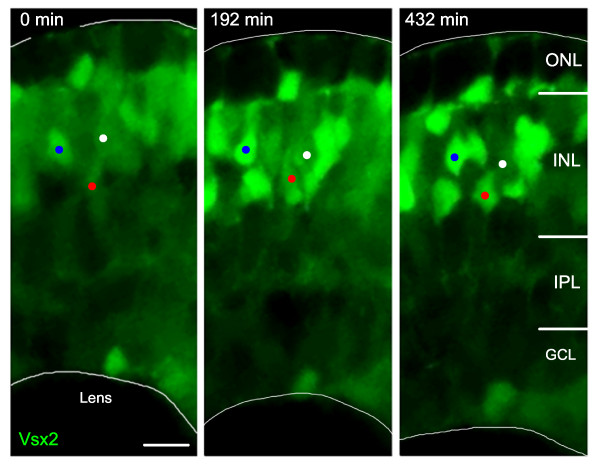
**Vsx2:GFP up- and downregulation**. Time-lapse images from Additional file 1 of a developing Tg(*vsx2:GFP) *retina. As development progresses, some cells (one example marked by a white dot) reduce their Vsx2:GFP expression, whereas other cells (two examples marked by blue and red dots) increase the expression of GFP and differentiate as bipolar cells with processes in the IPL. GCL, ganglion cell layer; INL, inner nuclear layer; IPL, inner plexiform layer; ONL, outer nuclear layer. Scale bar: 10 μm.

To investigate the *in vivo *role of Vsx2 in the zebrafish retina, we used Tg(*vsx2:GFP*) and Tg(*vsx2:dsRed*) transgenic zebrafish [26]. These transgenic lines have most of the regulatory regions of *vsx2 *driving the expression of cytoplasmic versions of these fluorescent proteins. To ensure that the green fluorescent protein (GFP) was faithfully reporting the activity of the endogenous *vsx2 *promoter, we compared the distribution and dynamics of the GFP monitored by fluorescence to the *vsx2 *RNA using *in situ *hybridisation in the developing retinas of Tg(*vsx2:GFP*) embryos. Consistent with a previous study, both *vsx2 *RNA andGFP are first detected in the optic primordium around 15/16 hpf [23]. At 24 hpf, a time when the retina consists of a neuroepithelium, *vsx2 *expression spreads throughout the retina, consistent with its expression in most if not all RPCs (Figure 1A, C). As retinal development progresses, some cells upregulate the expression of Vsx2:GFP, whilst others lose *vsx2 *RNA expression and GFP leading to a punctuate appearance of isolated labelled nuclei (Figures 1B, D and 2; Additional file 1).

In agreement with its role in bipolar cell fate in mouse, Vsx2:GFP becomes enriched in a population of bipolar cells in the outer part of the INL of 80 hpf retinas (Figure 1B, E–G). These cells can be identified as bipolars, as they have the morphology of bipolar cells with processes ending in the two plexiform layers. The vast majority of these remain negative for protein kinase C (PKC)β1, a marker for a subpopulation of bipolar cells (Figure 1E), demonstrating that Vsx2:GFP cells do not account for all bipolar cells, although a few do express PKC by 120 hpf (data not shown). Cells with lower levels of GFP expression are also seen in the more middle region of the INL. These cells have longer processes reaching apically into the outer nuclear layer (ONL) and basally to the end of the ganglion cell layer (GCL), and they all express both Sox2 and glutamine synthetase, markers of Müller glia [27]. Therefore, Vsx2 is also maintained (albeit at lower levels) in Müller cells (Figure 1F", G", asterisks). Both the GFP and *vsx2 *RNA are also present in the ciliary margin at 72 hpf (Figure 1B, D). In conclusion, similarly to the RNA, GFP expressed in the Tg(*vsx2:GFP*) line is present in all RPCs from around 16 hpf to around 28 hpf and later on becomes restricted to bipolar and Müller cells.

### Vsx1 and Vsx2 are expressed in distinct subsets of bipolar cells

**Figure 3:**
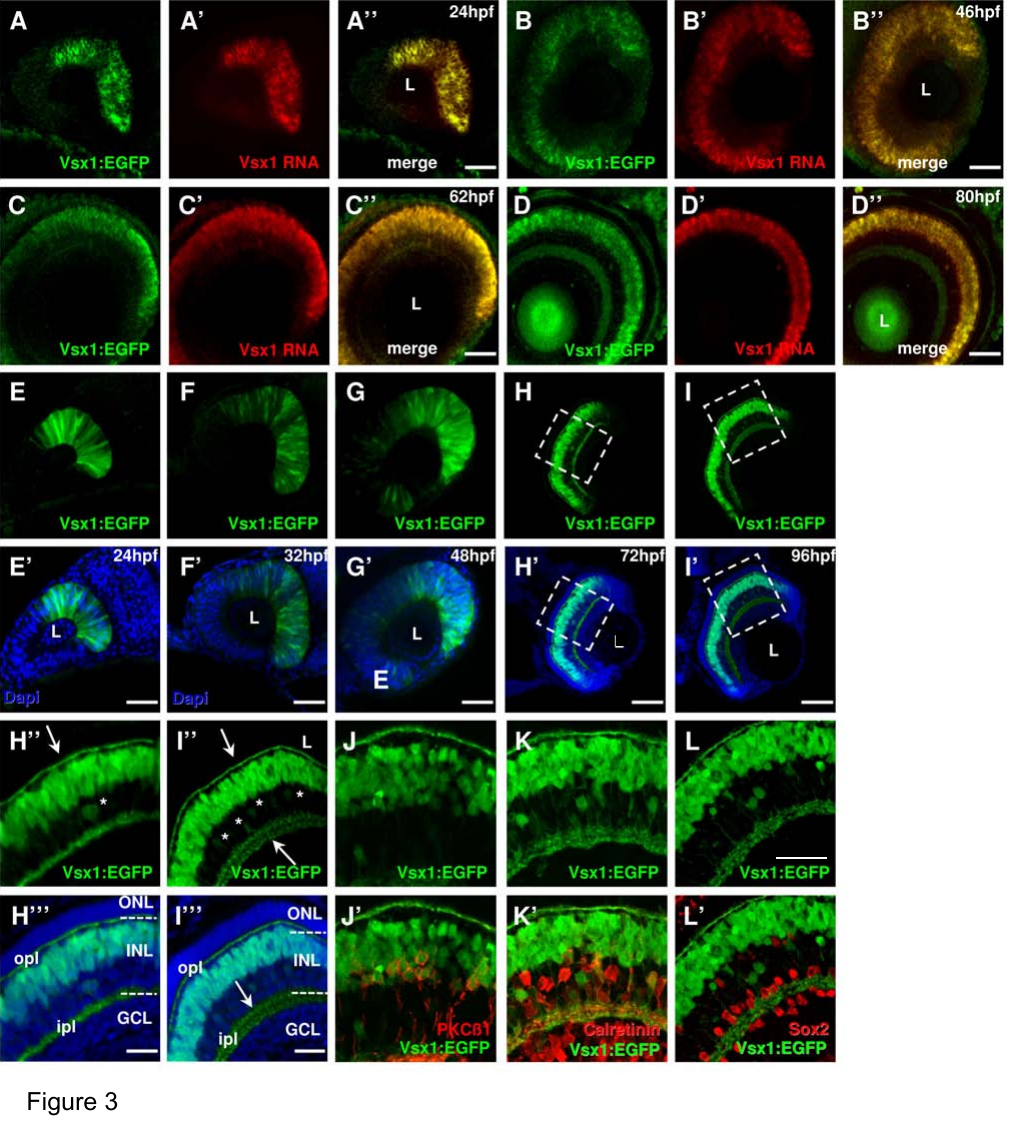
**Vsx1 expression in the zebrafish retina**. (A-D)*vsx1 in situ *hybridization in 24 hours post-fertilization (hpf) (A), 46 hpf (B), 62 hpf (C) and 80 hpf (D) Tg(*vsx1:GFP*) transgenic embryos shows complete co-localisation of *vsx1 *RNA and Vsx1 driven GFP expression. (E-I)Whole-mount lateral view of the Tg(*vsx1:GFP*) retina of zebrafish embryos counterstained with DAPI (blue) reveals expression of green fluorescent protein (GFP) in a subpopulation of retinal progenitor cells from 24 to 48 hpf as well as differentiated neurons at 72 and 96 hpf. (H-L)Cryosections through the central retina of Tg(*vsx1:GFP*) reveal GFP expression in cell bodies in the outer INL and processes in the OPL and IPL (arrows). GFP is also expressed in a few cells in the inner half of the INL (some indicated by asterisks). (J-L) Tg(*vsx1:GFP*) retinas at 80 hpf labelled with protein kinase C (PKC)β1 (J), calretinin (K) and Sox2 (L). Vsx1:GFP cells co-label with PKC-positive bipolar cells and calretinin-positive amacrine cells, but not with Sox2-labelled Müller cells. GCL, ganglion cell layer; INL, inner nuclear layer; ipl, inner plexiform layer; L, lens; ONL, outer nuclear layer; opl, outer plexiform layer. Scale bars: (A-I) 100 μm; (J-L) 40 μm.

Vsx1 is another homeobox protein of the paired-like class, and is also expressed in bipolar cells and is crucial for their differentiation [19,22-24]. We therefore examined Tg(*vsx1:GFP*) retinas, which express GFP under the control of *vsx1 *regulatory regions [28]. As with the Vsx2 transgenics, we found that the spatio-temporal expression pattern of GFP driven by the *vsx1 *regulatory sequences mirrors the expression of *vsx1 *RNA (Figure 3A–D). Vsx1:GFP is expressed in progenitors of the optic primordia, coming on slightly later though not as extensively as Vsx2, before being restricted to the INL (Figure 3E–I). Unlike Vsx2, however, no co-expression of Sox2 and Vsx1:GFP was observed in the differentiated retina, suggesting that Müller cells do not express Vsx1 (Figure 3L). Also unlike Vsx2, Vsx1 is expressed occasionally in amacrine cells (Figure 3H, I), some of which are positive for calretinin (Figure 3K). Thus, Vsx1 and Vsx2 are expressed in distinct subpopulations of non-bipolar cells.

**Figure 4:**
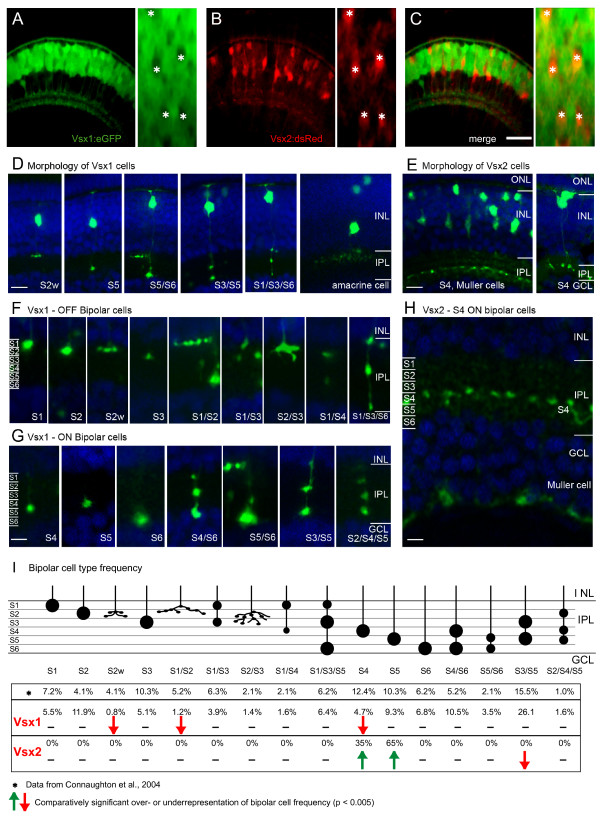
**Vsx1 and Vsx2 are expressed in the distinct subtypes of bipolar cells**. (A-C) Red and green fluorescence images from Tg(*vsx1:GFP*;*vsx2:dsRed*) double transgenic embryos, showing that they are expressed in separate populations of cells in the outer INL. Vsx2:dsRed positive neurons are Vsx1:GFP negative (asterisks) (D-H)Confocal images showing the morphology of individual Vsx1:GFP (D, F, G) and Vsx2:GFP (E, H) cells. Counterstained with DAPI (blue). Morphology of entire Vsx1 (D) and Vsx2 (E) expressing cells reveals bipolar cells are labelled. (F-G) Higher magnification of the IPL reveals different subtypes identified by their axon terminal stratification depth and pattern. Vsx1:GFP is expressed in many types of OFF bipolar (F) and ON bipolar (G) subtypes, which stratify throughout the entire IPL. In contrast, Vsx2:GFP is expressed in only one type of bipolar cell stratifying in stratum 4 (E, H) and in Müller cells, whose endfeet can be seen in the GCL. (I)Schematic showing the axon terminals and frequency of occurrence of Vsx1:GFP and Vsx2:GFP bipolar subtypes together with the previously described frequency from diOlistic labelling [29]. Three types of Vsx1:GFP cells occur at a statistically lower frequency than described previously and may be underrepresented in the Vsx1:GFP-labelled population (red arrows). The two types of Vsx2:GFP cells (S4 and S5), occur at a statistically higher frequency in the Vsx2:GFP population than the diOlystic population (green arrows). GCL, ganglion cell layer; INL, inner nuclear layer; IPL, inner plexiform layer; ONL, outer nuclear layer. For quantification of subtypes, IPL was subdivided into six equal strata (S1–S6). Scale bars: (A-C) 40 μm; (D, E) 10 μm; (F-H) 5 μm.

Do Vsx2 and Vsx1 also label distinct subpopulations of bipolar cells? Vsx1 labelled many more bipolars than Vsx2, including PKCβ1-positive cells (Figure 3J). To test if any bipolar cells expressed both Vsx1 and Vsx2, we crossed Tg(*vsx2:dsRed*) with Tg(*vsx1:GFP*) fish to create double transgenic embryos. At 96 hpf, zebrafish retinas of double trangenic GFP (Vsx1) or DsRed (Vsx2) cells were separated into distinct populations, as revealed by the lack of co-labelling (Figure 4A–C). Vsx1:GFP unlabeled cells within the bipolar cell layers are usually labelled with Vsx2:DsRed, and vice versa, showing that virtually all bipolar cells are either Vsx1 or Vsx2-positive.

To better characterize the bipolar cell types, we performed injections of *vsx1:GFP *bacterial artificial chromosomes (BACs; and/or transplantations of transgenic cells to wild-type embryos) so that individual cell morphology could be observed. These two techniques gave similar results. Our morphological classification of the GFP expressing cells shows that Vsx1 is expressed in a vast variety of bipolar subtypes (Figure 4D). Based on soma position, axon terminal morphology and stratification, 17 different types of bipolar cells have been characterized in the zebrafish retina using DiOlistic techniques [29]. All of the described bipolar subtype cells were encountered in our sample of 514 Vsx1:GFP-positive cells (Figure 4F, G). However, the frequency of two subtypes with axon terminals in the outer inner plexiform layer (IPL) and one with axon terminals in the inner IPL was found to be significantly lower (binomial test, *p *< 0.005) than the frequency reported by Connaughton *et al*. [29], suggesting that Vsx1 may not be expressed evenly in all bipolar subtypes (Figure 4I). The same analysis on Vsx2:GFP bipolars showed that Vsx2-positive axon terminals are located in the inner half, which is also referred to as the ON sublamina of the IPL. Therefore, in contrast to Vsx1, Vsx2 is expressed in only one or two bipolar cell subtypes. Indeed, axon terminals were exclusively found between stratum 4 or 5 (based on six equal subdivisions of the IPL depth). Based on the regular spacing and consistent laminar stratification of Vsx2 terminals (Figure 4E, H), we believe that these cells make up a single type, probably the S4 bipolar type, as it was one that occurred at a significantly lower frequency in our Vsx1 sample (Figure 4I). Thus, Vsx2 bipolar cells are largely or entirely distinct from the Vsx1-positive ones. These results showing mutual exclusion in bipolar cells supports recent findings that Vsx2 acts as a repressor of *vsx1 *transcription [20].

### Vsx1 bipolar cells arise from progenitors that turn off Vsx2

**Figure 5:**
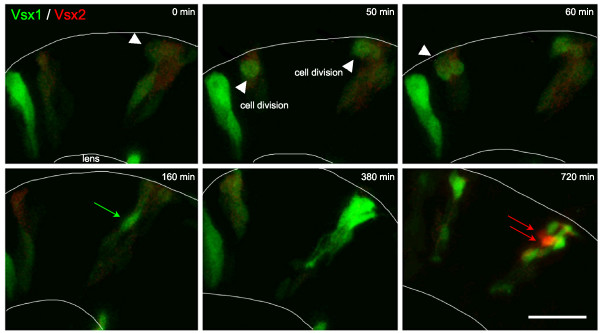
**Vsx2 versus Vsx1 arise from distinct lineages**. Time-lapse images from Additional file 2 showing cells transplanted from Tg(*vsx1:GFP; vsx2:dsRed*) into wild type to reveal individual cell morphology. Vsx1:GFP progenitors that have already lost Vsx2:DsRed (green only) or are in the process of (weak red signal remaining in these green cells) downregulating Vsx2:DsRed divide apically (white arrowheads). They upregulate Vsx1:GFP (green arrows) and differentiate as a group of bipolar cells. Vsx2:DsRed is expressed at a low level initially. It fades in cells that turn on Vsx1:GFP, but the red signal also fades during the movie shown in Additional file 2 due to bleaching, so Vsx2:DsRed progenitors cannot be followed throughout the entire movie. Vsx2:DsRed is later dramatically upregulated in cells that do not express Vsx1 (red arrows). Scale bar: 20 μm.

**Figure 6:**
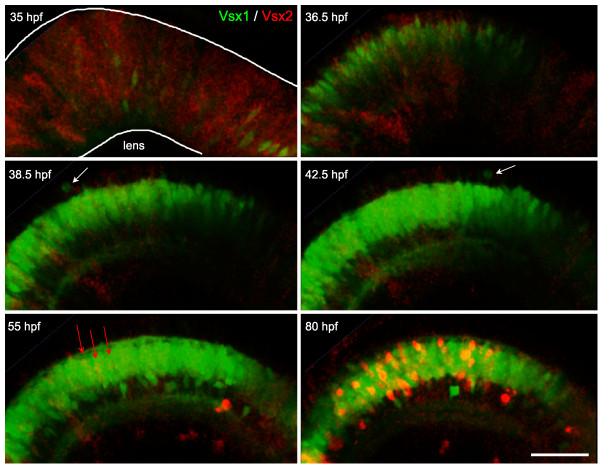
**Vsx2/Vsx1 down-/upregulation, followed by Vsx2 upregulation**. Time-lapse images from Additional file 3 of a double transgenic Tg(*vsx1:GFP; vsx2:dsRed*) embryo. Vsx2 is initially expressed in the majority of progenitors in the developing neuroepithelium and becomes downregulated in cells that upregulate Vsx1. These cells continue to undergo cell divisions at the apical surface (white arrows) and start differentiating around 40 hours post-fertilization (hpf). Later (around 55 hpf), a subpopulation of cells starts upregulating the expression of Vsx2:DsRed (red arrows) and then differentiates in the inner nuclear layer. Scale bar: 500 μm.

To investigate how these two bipolar populations are generated, we made time-lapse movies on transgenic embryos. Vsx2-positive bipolar cells were seen to arise from Vsx2-positive progenitors (Figure 2; Additional file 1). After the last division of such a progenitor, Vsx2:GFP expression increases dramatically as the bipolar cells move to their final position in the INL and begin to differentiate. In double transgenic embryos, Tg(*vsx2:dsRed*;*vsx1:GFP*), red 'Vsx2-positive' bipolar cells generally arise from progenitors that are 'Vsx1-negative', that is, not green (Figure 5; Additional file 2). Vsx1 expressing bipolar cells, however, initially arise from progenitors that express Vsx2 (Figure 6). However, as soon as Vsx1 expression begins in these progenitors, Vsx2 expression decreases (Figure 6; Additional file 3). Some, and perhaps all, of these Vsx1-positive, Vsx2-negative cells go through at least one round of mitosis before they differentiate (Figures 5 and 6; Additional files 2 and 3). Thus, Vsx2 bipolar cells arise from Vsx2+/Vsx1- progenitors. In contrast, Vsx1-positive progenitors that have downregulated Vsx2 divide to produce Vsx1-positive bipolar cells.

**Figure 7:**
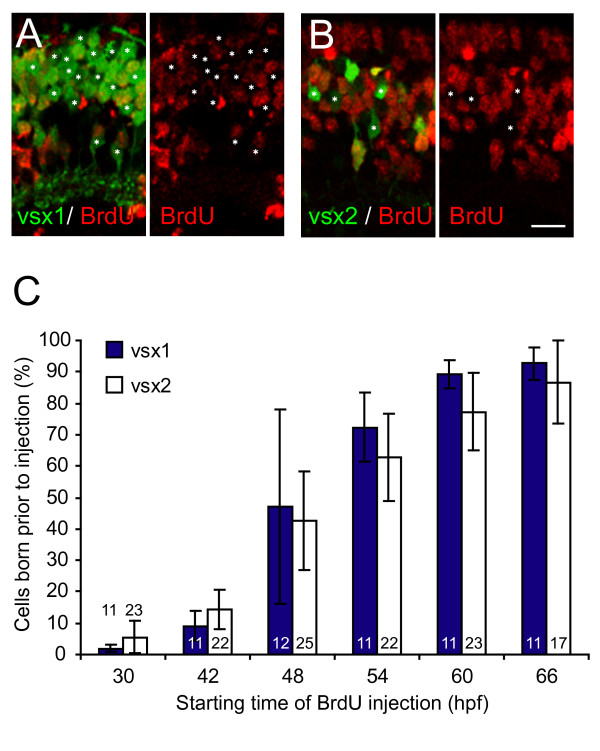
**Vsx1 and Vsx2 bipolar subtypes are born at the same time**. Quantification of cumulative bipolar birthdates in bromodeoxyuridine (BrdU)-injected Tg(*vsx1:GFP*) and Tg(*vsx2:GFP*) retinas. (A, B) Micrographs of central sections of retina through 75 hours post-fertilization (hpf) transgenic zebrafish showing Vsx1:GFP (A) or Vsx2:GFP (B) expression and BrdU immunohistochemistry. Prior to BrdU injection at 42 hpf, about a third of Vsx1:GFP- and Vsx2:GFP-positive cells in this central region of the retina underwent terminal division and are, therefore, BrdU immunonegative (asterisks). (C) Percentage of GFP-positive cells from Tg(*vsx1:GFP) *or Tg(*vsx2:GFP*) at 75 hpf that were born prior to the onset of BrdU injections. The temporal birth pattern of Vsx1:GFP cells and Vsx2:GFP cells is the same. Most of the vsx1 and vsx2 GFP-positive cells are born between 42 and 60 hpf. Numbers in bars indicate number of sections analysed. For Vsx1:GFP, the total number of cells included for each time point ranged from 1,209 to 1,864; for Vsx2:GFP, the total number of cells included ranged from 820 to 1,226. Note that the measurements in this graph include the entire retina (that is the central retina shown in (A, B) and the more peripheral regions where cells are born later), leading to a lower percentage in the graph than shown in the micrographs in (A, B), which are taken of the central section. Error bars show standard deviations. Scale bar: 10 μm.

Vsx1-positive bipolars begin to differentiate at 40–42 hpf, suggesting that they may be the first bipolar cells to be born in the retina (Figure 6; Additional file 3). Vsx2 bipolar cells start to be recognized 15 hours later at 55–60 hpf (Figure 6; Additional file 3). This suggests that the two populations might be generated at different times. Analysis of 75 hpf retinas of embryos injected every 12 hours with BrdU from 30 hpf onwards, however, showed no difference in the timing of the terminal divisions of the Vsx1- and Vsx2-positive bipolar types (Figure 7). We therefore suggest that Vsx2 bipolar cells take a longer time to differentiate into recognizable subtypes than Vsx1 bipolars, remaining as neuroepitheial shaped cells, though both Vsx1- and Vsx2-expressing bipolar cells are born within the same temporal window.

### Progenitors of other lineages also turn off Vsx2

**Figure 8:**
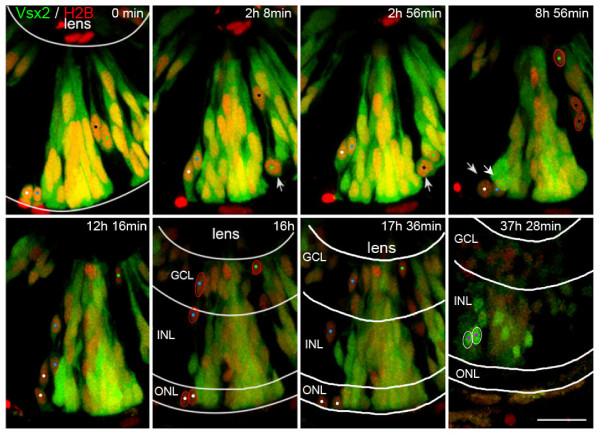
**Some Vsx2 progenitors lose Vsx2 expression and differentiate as presumed photoreceptors, amacrine cells and ganglion cells**. Time-lapse images of Additional file 4, showing transplanted cells from a transgenic Tg(*vsx2:GFP) *embryo that was injected with *H2B:RFP *RNA to mark all cells transplanted. Initially, all transplanted progenitor cells express Vsx2. Some cells (white arrows) undergo cell division and downregulate Vsx2:GFP expression (marked by white arrows at mitosis and white, blue, green and black spots). The Vsx2-negative daughter cells of these divisions (red outlines) end up in the ONL (presumed photoreceptors), inner INL (presumed amacrine cells) and GCL (presumed ganglion cells). In contrast, cells that upregulate the Vsx2:GFP expression late during development become restricted to the INL only, where they differentiate into Vsx2-positive bipolar or Müller cells (purple dots, white outlines). GCL, ganglion cell layer; INL, inner nuclear layer; ONL, outer nuclear layer. Scale bar: 20 μm.

As Vsx2 appears to be downregulated in most RPCs, we decided to monitor Vsx2 progenitors during retinal differentiation. To see all the descendents of the Vsx2 RPCs, Tg(*vsx2:GFP*) transgenic embryos were injected with *H2B*:*RFP *RNA to label nuclei. Cells from such injected embryos were then transplanted into unlabelled wild-type embryos and imaged every 8 minutes for 40 hours from 30 hpf onwards. In these movies, individual Vsx2:GFP RPCs can be followed. Initially, all the transplanted cells expressed Vsx2:GFP and H2B:RFP. Some of these progenitors are then seen to diminish their GFP while keeping RFP as they go through cell division near the apical surface (Figure 8; Additional file 4). The resulting RFP descendents are also progenitors in that they undergo typical interkinetic nuclear migration and divide apically (Figure 8; Additional file 4). The daughters of these RFP progenitors then become restricted to particular positions in the retina (red outlines in Figure 8). Some of these come to reside in the GCL (presumably becoming ganglion cells), some take up residence in the INL, including the inner part of the INL (possibly amacrine cells), and some come to lie with photoreceptors in the ONL (Figure 8). As the RFP is a nuclear marker, it did not allow us to determine cell type by morphology, but the fact that these nuclei are no longer going through interkinetic migration or dividing leads us to believe that these cells are differentiating into the various cell types other than Vsx2-positive bipolars or Müller cells. In contrast, cells that retain and then upregulate the expression of Vsx2:GFP move to their final position in the INL, where the expected Vsx2-bipolar or Müller cells are found (Figure 8).

### Ath5 is a potential target of Vsx2 repression

The above results suggest that Vsx2 may repress all lineages in the retina except for the lineage leading to S4 bipolars and Müller cells. This seems to be the case for Vsx1-positive bipolars as Vsx2 represses Vsx1 [20] and is turned off in the Vsx1-positive progenitors that will give rise to all other bipolar cell types. To investigate the hypothesis that Vsx2 represses other lineages, we took advantage of the Tg(*ath5:RFP*) transgenic lines [30,31]. Ath5 is a bHLH transcription factor expressed in some retina progenitor cells and essential for the determination of the retinal ganglion cell fate [32]. Poggi *et al*. [30] demonstrated that Ath5 is turned on in progenitors before their terminal division at about 28 hpf. Ath5-positive progenitors generally divide once and one daughter of this division then becomes a ganglion cell, while the other daughter remains more apical, often becoming a photoreceptor. In Tg(*ath5:GFP*) embryos, one can see that half of the GFP-positive cells in the retina at 96 hpf are RGCs while the other half are a mixture of amacrine cells, horizontal cells and photoreceptors (both rods and cones) [30], suggesting that Ath5 progenitors give rise to all these cell types. Ath5-positive bipolars and Müller cells were rarely if ever seen [30], suggesting that these cell types arise from different lineages, which we now know to be the Vsx1 and Vsx2 lineages.

**Figure 9:**
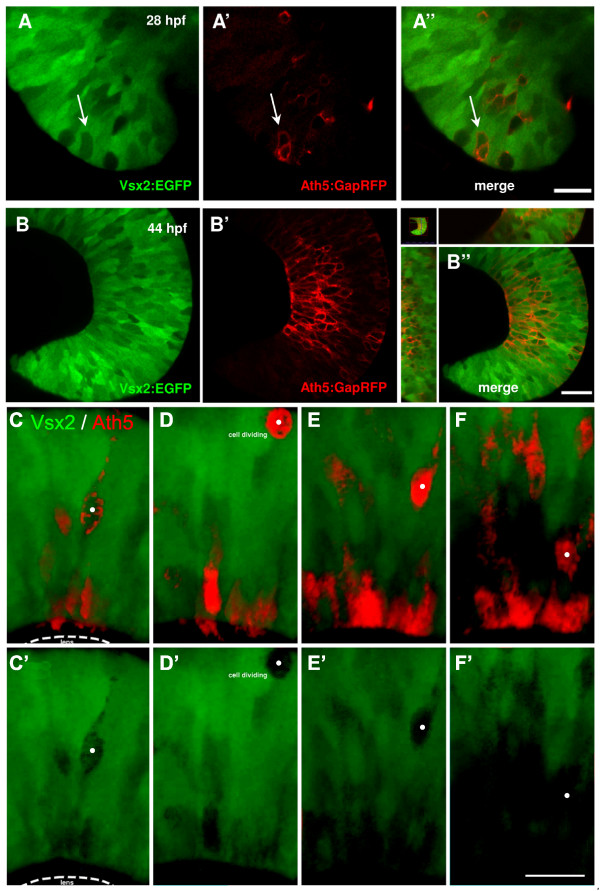
**Ath5 progenitor cells arise from Vsx2 progenitors that lose Vsx2:GFP expression**. (A, B) Images of live double transgenic Tg(*vsx2:GFP/ath5:GapRFP*) whole-mount retinas at different time points of retina development indicated as hours post-fertilisation (hpf). (A) At 28 hpf, a few cells in the retina no longer express green fluorescent protein (GFP) driven by *vsx2*. Some (white arrows), but not all of these Vsx2:GFP-negative cells express Ath5:RFP. (A'-B') As development progresses, the number of cells that express Ath5:RFP increase as the number of Vsx2:GFP cells diminishes. Ath5-expressing, Vsx2:GFP-negative cells divide apically and many of the differentiating cells can be seen to settle in the developing ganglion cell layer, which becomes devoid of Vsx2:GFP cells. Images are from the movie in Additional data file 5. (C-F) Double label from a Tg(*ath5:RFP;vsx2:GFP*) retina in which an Ath5:RFP progenitor divides once to produce one daughter that differentiates as an RGC. (E'-F') Vsx2 expression can be seen to be downregulated in this Ath5:RFP progenitor (whose soma is marked by the white dot). Scale bars: (A) 16 μm; (B) 27 μm; (C-F) 20 μm.

If Ath5 progenitors arise from Vsx2 progenitors, then we should expect to see Vsx2 downregulation coordinated with Ath5 upregulation in Tg(*vsx2:GFP;ath5:GAP-RFP*) double transgenics. Strikingly, time-lapse analysis of the developing retina reveals that RFP driven by *ath5 *is first detected in 'holes' that open in the Vsx2:GFP expression domain (Figure 9A–B"). Indeed, Vsx2:GFP and Ath5:RFP were never found to be co-expressed, demonstrating that Ath5 is restricted to progenitors that have lost Vsx2 expression. Ath5 progenitors that no longer express Vsx2 can be seen to divide apically and settle in their final position in the ganglion cell layer, which becomes devoid of Vsx2:GFP-labelled cells (Figure 9C–F'; Additional file 5). While a significant fraction of the 'holes' are Ath5:RFP-positive, there appear to be at least as many holes that are Ath5:RFP-negative (Figure 9A–B"). Cells that downregulate Vsx2 might also, therefore, be other kinds of restricted progenitors, such as, for example, Vsx1-positive progenitors.

The absence of Ath5 expression in Vsx2 expressing cells together with the close parallel of Ath5 onset and Vsx2 downregulation suggest that Vsx2 could directly regulate *ath5 *expression as it does *vsx1 *[20]. Indeed, previous work demonstrates that Vsx2 can bind to several different kinds of promoters, whose genes are derepressed in the absence of Vsx2 [11]. We used the optimal Vsx2 target sequence PyTAATtPuPu identified in that study to look for putative Vsx2 DNA binding sites present in 3-kb upstream regulatory regions of zebrafish *ath5*. A consensus sequence can be found at position -270 bp in the zebrafish *ath5 *upstream region.

**Figure 10:**
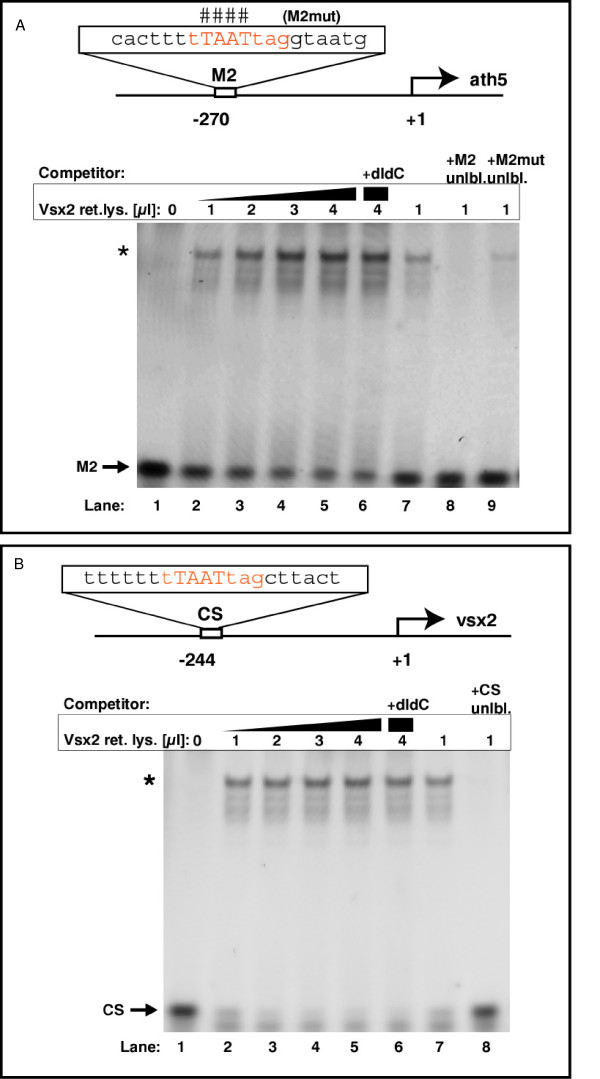
**Vsx2 binds to *ath5 *and *vsx2 *promoter sequences *in vitro***. (A)*In vitro *translated Vsx2 protein was incubated with a Cy5-end-labeled probe against the *ath5 *promoter fragment (M2) with or without competitors. The binding sequence core of M2 is shown in red. Lanes 2–5: addition of increasing amounts of Vsx2 protein shifts cumulative amounts of M2 oligo (asterisk; band shift). *In vitro *translated luciferase protein served as negative control and induced no band shift (lane 1). Addition of the unspecific competitor poly(dI-dC) (lane 6) did not reduce binding. Lanes 7–9: band shift of labelled M2 oligo by Vsx2 was challenged by adding specific competitors. Addition of excess unlabeled M2 oligo led to out-competition of the labelled M2 oligo (lane 8 versus lane 7). Addition of excess unlabeled mutated M2 oligo could compete only weakly with binding (lane 9). The bases mutated in this oligo are indicated by the hash symbols above the binding sequence core. (B) *In vitro *translated Vsx2 protein was incubated with a Cy5-end-labeled probe against the *vsx2 *promoter fragment (CS) with or without competitors. The binding sequence core of CS is shown in red. Lanes 2–5: addition of increasing amounts of Vsx2 protein shifts cumulative amounts of CS oligo (asterisk; band shift). *In vitro *translated luciferase protein served as negative control and induced no band shift (lane 1). Addition of the unspecific competitor poly(dI-dC) (lane 6) did not reduce binding significantly. Lanes 7–8: addition of excess unlabeled CS oligo led to out-competition of the labelled CS oligo.

We checked to see if Vsx2 was capable of binding to this site using an electrophoretic mobility shift assay (EMSA) with oligos containing the candidate sequence using *in vitro *synthesized Vsx2 protein. Indeed, Vsx2 binds specifically to the site at -270 bp found in the *ath5 *promoter in a concentration-dependent manner, but does not bind to mutant oligos in a competitive assay (Figure 10A).

### Vsx2 affects bipolar cell and other fates

**Figure 11:**
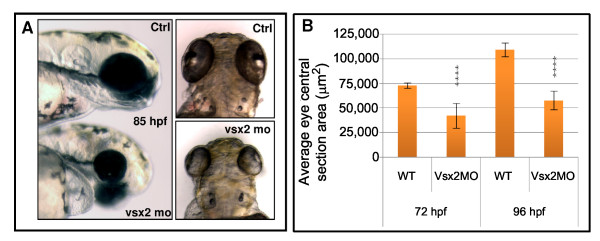
**Vsx2 depletion leads to microphthalmia in zebrafish**. (A) Lateral and dorsal views of representative 85 hours post-fertilization (hpf) control (Ctrl) and Vsx2 morphant (mo) embryos. Vsx2 morphants are microphthalmic. (B) Graph showing the area of central retina sections of control (wild type (WT)) and Vsx2 morphant (Vsx2MO) at 72 hpf and 96 hpf; 10–20 sections from different eyes were analysed. Vsx2 morphant retinas show a significant (asterisks) reduction in eye area (*p *< 0.0001). Error bars indicate Standard Error of the Mean.

**Figure 12:**
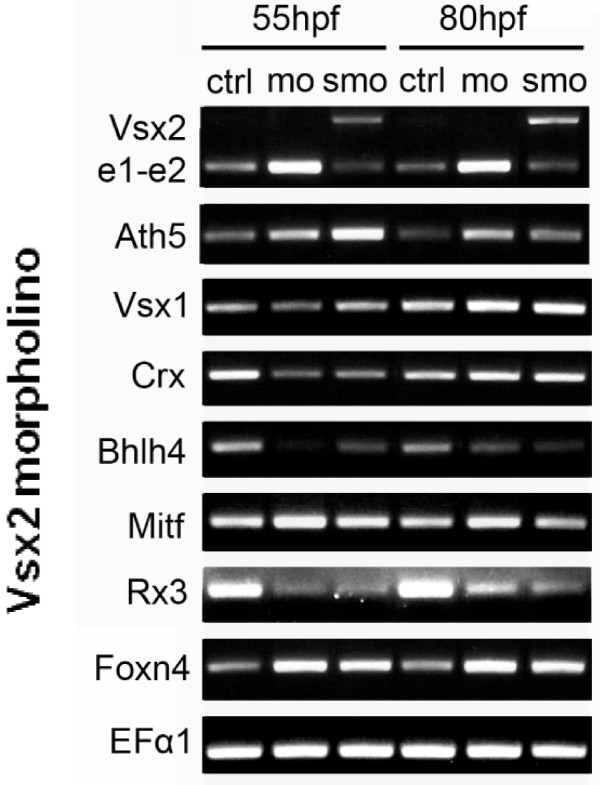
**Transcription factors that are upregulated and downregulated in Vsx2 morphants**. RT-PCR of control (Ctrl) and Vsx2 morphant embryos showing changes in gene expression. mRNAs for Vsx2, Ath5, Mitf, and Foxn4 are all upregulated in Vsx2 morphants, suggesting that these transcription factors are normally repressed by Vsx2. On the other hand, Crx, Bhlh4 and Rx3 are downregulated in Vsx2 morphants, suggesting that these genes are indirectly positively controlled by Vsx2. Vsx1 levels seem to be unchanged in Vsx2 morphants. Vsx2e1-e2 (the PCR product of primers that amplify the Vsx2 region from exon 1 to exon 2) is also upregulated when Vsx2 is knocked down with the translational morpholino (mo), whilst efficiency of the splicing morpholinos (smo) can be seen by the increased PCR product size due to splicing being prevented by the morpholino. The housekeeping gene encoding EF1α (Elongation factor 1α) was used as a loading control.

**Figure 13:**
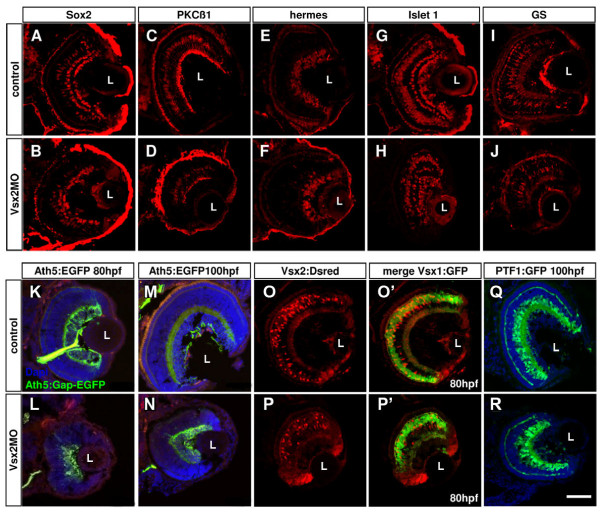
**Loss of Vsx2 allows various cell types to develop in the retina**. (A-J) Cryosections of 80 hours post-fertilization (hpf) retinas labelled with different cell-specific markers show that Vsx2 morphant retinas have all cell types present: Sox2 labels amacrine and Müller cells (A, B); protein kinase C (PKC)β1 labels some bipolar cells (C, D); Hermes labels ganglion cells (E, F); Islet 1 labels horizontal, bipolar, some amacrine and ganglion cells (G, H); glutamine synthetase (GS) labels Müller cells (I, J). (K-T)Cryostat sections of 80 hpf transgenic retinas similarly reveal presence of all marked cell types in Vsx2 morphants. (K-N) Vsx2 morphant Tg(*ath5:Gap-GFP*) retinas (double labelled with the ganglion cell marker Zn5 in red) still have green fluorescent protein (GFP)- and Zn5-labelled ganglion cells and form an optic nerve to the tectum. (O-R) Vsx2 morphant double transgenic Tg(*vsx1:GFP-vsx2:dsRed*) have both GFP- and DsRed-labelled cells, but appear to have comparatively fewer Vsx2:DsRed cells. As in the control transgenics, Vsx1:GFP and Vsx2:DsRed do not co-localise in the same cells. (S, T) Vsx2 morphant Tg(*ptf1a:GFP*) retinas show GFP labelling for horizontal and amacrine similar to that in controls, although some GFP cells are displaced and can be found closer to the lens in the GCL of the morphant retinas. L, lens. Scale bar: (A-T) 47 μm.

To assess the role of Vsx2 in cell determination, we used either the translation blocking morpholino (MO) or a combination of 2 splicing MOs (SMOs) affecting the splicing in introns 1 and 2, respectively, to knock down Vsx2. It was necessary to inject the two SMOs together to abrogate normal splicing of Vsx2. Morphant embryos, from the translation blocker or the SMOs, are clearly microphthalmic (Figure 11A, B), in accord with what has been previously recorded [25]. We observed changes of expression of several genes in Vsx2 morphants (Figure 12). Comparable results were found when comparing the expression levels of these genes using RNA from only morphant heads or even only eyes (data not shown). As expected from the finding that Vsx2 can bind the *ath5 *promoter, we found an upregulation of *ath5*, which fits well with our finding that Ath5:RFP expression is negatively correlated with Vsx2 expression and that Vsx2 binds the *ath5 *promoter *in vitro*. *foxn4*, a gene that has been described as necessary for the mitotic progenitors of amacrine and horizontal cells in the mouse retina [33] (Figure 12), is also upregulated in Vsx2 morphants. As expected, from previous work, *mitf*, a retinal pigment epithelium -specific gene normally repressed by Vsx2, was also increased [13], while the bipolar-cell-specific gene *bhlh4*, on the other hand, was downregulated [34]. *rx3*, a gene whose late period of expression in Medaka fish is in the INL [35], was also downregulated (Figure 12). Surprisingly, there is a strong increase of *vsx2 *transcription itself in *vsx2 *morphants, suggesting that Vsx2 represses its own expression. In agreement with this, we found Vsx2 binding consensus sequences in the *vsx2 *regulatory element, and this element also interacts with Vsx2 in an EMSA assay (Figure 10B). Surprisingly, there is no clear effect on the level of Vsx1 expression at any time point analysed from 31 to 80 hpf (Figure 12). Although the eyes were clearly microphthalmic and poorly formed, ganglion cells (Hermes- and Islet-positive), amacrine cells (Sox2- and 5E11-positive; Ptf1:GFP cells in the inner part of the INL), horizontal cells (Ptf1:GFP cells in the outermost part of the INL near the outer plexiform layer (OPL)), cone and rod photoreceptors (Zpr1- and 3-positive, respectively, data not shown) were detected in Vsx2 morphants (Figure 13A–J). Both Müller glial cells (glutamine synthetase-positive) and PKC-positive bipolars appeared reduced in numbers at 72 hpf (Figure 13D, J). Indeed Vsx2 morphant Tg(*vsx2:dsRed;vsx1:GFP*) double transgenic embryos showed a reduced density of dsRed compared to GFP-labelled cells in the INL (Figure 13O–R).

**Figure 14:**
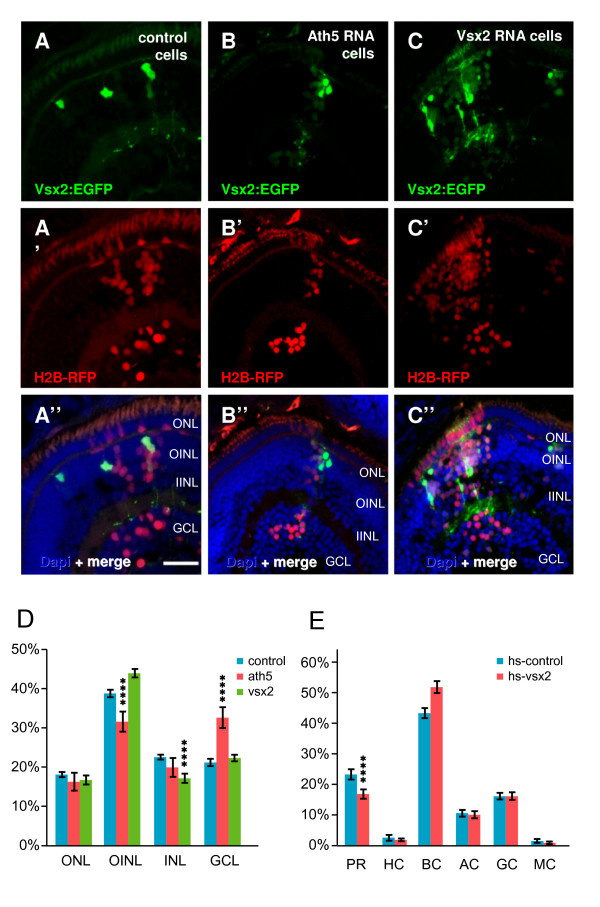
**Transplantation of wild type, Ath5, and Vsx2 overexpressing cells in wild-type embryos**. (A-C)Tg(*vsx2:GFP*) transplanted into wild-type embryos after injection with *H2B-RFP *RNA (to mark all transplanted cells) and various constructs for overexpression. (A) Green-fluorescent protein (GFP)-expressing cells from control Tg(*vsx2:GFP*) become bipolar and Müller cells, whilst all (H2B-RFP-positive) cells become all kinds of cells in the expected frequency. (B) When co-injecting *H2B-RFP *RNA with *ath5*, the ganglion cell fate is promoted (more H2B-RFP cells in the GCL), but Vsx2:GFP cells still become bipolar and Müller cells. (C) Co-injection of *H2B-RFP *RNA with *vsx2 *promotes bipolar cell fate, seen by (D) a highly significant (p < 0.001) increase of H2B-RFP in the INL bipolar layer at the expense of amacrine cells (asterisks). (E) Overexpression using a heatshock *vsx2 *construct similarly results in an increased frequency of bipolar cells, this time at the expense of photoreceptors. Error bars indicate Standard Error of the Mean. AC, amacrine cell; BC, bipolar cell; GCL, ganglion cell layer; H, horizontal cell; hs, heat shock; IINL, inner half of inner nuclear layer; MC, Müller cell; OINL, outer half of inner nuclear layer; ONL, outer nuclear layer; PH, photoreceptor; RGC, retinal ganglion cell. Scale bar (A-C) 25 μm.

For misexpression, we found that injection of *vsx2 *mRNA led to embryos that were anophthalmic, making such an analysis problematical. To get around this problem, we either transplanted cells from Vsx2 misexpressing Tg(*vsx2*:GFP) embryos into wild-type hosts or injected embryos with the *pSGH:vsx2 *heatshock plasmid and then incubated the injected embryos at 39°C from 26–28 hpf. Given that a decrease in Vsx2 interferes with bipolar fates in favour of other cell types, we wanted to test whether Vsx2 misexpression promotes bipolar fate at the expense of other cell types. Analysis of the resulting fate change was done on 80–85 hpf retinas. In the transplant experiments, *H2B:RFP *mRNA was co-injected to compare the proportion of each cell type present (Figure 14A–C). Ath5, which promotes ganglion cell fate, was used as a positive control and, indeed, *ath5 *RNA injections resulted in an increase in the ganglion cell population. In *vsx2 *RNA injected cells, we observed an increase in cells in the bipolar layer (outer INL) at the expense of cells in the inner INL (Figure 14D). In the heatshock overexpression experiments, there was an increase in the bipolar cells, but this time at the expense of photoreceptors (Figure 14E).

## Discussion

The transcription factor Vsx2 is initially expressed in all RPCs of the zebrafish optic vesicle, but then turns off in the progenitors of most cell types. Some bipolar cells upregulate Vsx2, but the majority of differentiated retinal cells are Vsx2-negative. Indeed, most bipolar cells are also Vsx2-negative, expressing the closely related Vsx1 instead. Time-lapse imaging shows that Vsx1 is upregulated only in RPCs that downregulate Vsx2, consistent with the data in mice showing that Vsx2 represses *vsx1 *[20]. Vsx2 is expressed only in the S4 or S5 types of bipolar cell, while the closely related Vsx1 is expressed in all 17 previously characterized zebrafish bipolar cells [29], but with a reduced frequency in the S4 bipolar cell type. Taking into account the small degree of inaccuracy when subdividing the IPL (which may result in occasional misclassification of bipolar cells), and considering the even spacing and high frequency of the Vsx2 expressing bipolar cells, we must consider the possibility that Vsx2 is expressed in an entire population of the S4 bipolar cell subtype, which does not express Vsx1. S4 bipolar cells are one of 8 ON-type bipolar cell types. They have a single terminal bouton stratifying in the inner IPL. Their dendritic tree is of distal comb or flat morphology, suggestive of rod or mixed photoreceptor input [36]. In other species, Vsx1 and Vsx2 are also expressed in mutually exclusive populations of bipolar cells. In the mouse, Vsx1 is expressed by different types of ON and OFF cone bipolar cells, whereas Vsx2 is expressed in other types of cone and rod bipolar cells [8,19,22,37]. Consistently, Vsx1 mutations lead to a reduction in bipolar-specific immunohistochemical markers and to defects in OFF-bipolar (mice) or ON-bipolar (human) driven visual responses, with normal rod bipolar driven scotopic responses [19,22,37-39].

**Figure 15:**
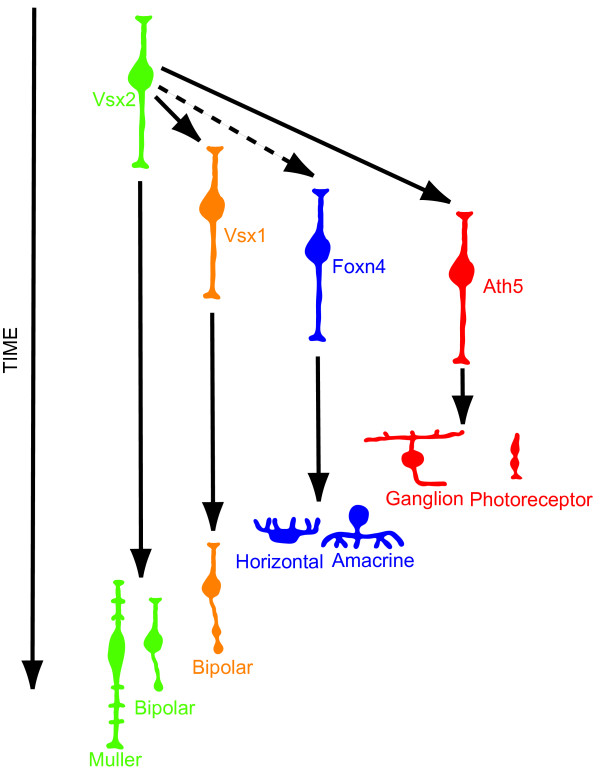
**Proposed model for Vsx2 as a cell fate time regulator**. We propose that Vsx2 expressed in progenitors represses genes required for differentiation of multiple lineages, including Vsx1- and Ath5-expressing cells, and possibly also represses itself. Only when Vsx2 is turned off can cells proceed to initially form progenitors that express Vsx1 and later Ath5 (arrows). These cells will continue differentiation in the previously described conserved histogenic order. We show a possible repression of Ath5, the first gene known to be directly involved in neural cell fate during retina development. Our data also support the idea of a repression of horizontal and amacrine fate genes, possibly through Foxn4 (dashed arrow).

In our time-lapse movies of the development of Tg(*vsx1:GFP; vsx2:dsRed*), we see that Vsx1:GFP is turned on in a subset of cells that downregulate Vsx2:DsRed. This switch occurs early enough in the lineage that Vsx1:GFP cells remain dividing, consistent with the relatively late birthdates of bipolar cells. The Ath5 sublineage, which also derives from cells that turn off Vsx2, gives rise to RGCs and other cells that have earlier birthdates. This suggests a model of RPC fate restriction, in which progenitors become restricted in competence before their final cell divisions (Figure 15). In this model, Vsx2-positive RPCs are fully multipotent RPCs that throw off distinct sublineages at different stages of development (Figure 15). Some of these lineages, though arising earlier in development, have more proliferative potential than some other lineages that arise later. Indeed, Vsx1 expression comes on earlier in development than Ath5 expression, even though the birthdates of bipolar cells tend to be later than the birthdates of RGCs. Note that this model is somewhat different from the more standard model of retinogenesis [40] in which there is progressive fate restriction in the fates of a single population of RPCs (although Vsx2-negative lineage-restricted progenitors still have to go through a temporal sequence of neurogenesis). The two models are essentially different because one suggests that all progenitors are the same and that they progressively change over time, whereas the other suggests that selected progenitors (that is, Vsx2-negative ones) become lineage-restricted at early stages. This new model of fate restriction through derepression may help explain why there are general trends rather than a strictly conserved order of histogenesis in the vertebrate retina. This model may also explain why cell cycle inhibition does not result in a greatly imbalanced cell fate distribution [41]: if, for example, a Vsx1-positive bipolar lineage is specified early, then these cells might come out of the cell cycle early when the cell cycle is experimentally inhibited, but still become bipolars. Future time-lapse experiments may resolve such issues, but this will require triple or even quadruple colour transgenics where daughter cells of such progenitors, expressing markers of particular sublineages from the same parent Vsx2-positive cell, can be individually followed. It will also be important to understand why different Vsx2-negative progenitors turn on distinct lineage-specific transcription factors.

We tracked Vsx2 expression by following fluorescent proteins (that is, GFP or dsRed) under the control of the Vsx2 promoter, and we therefore expected that the perdurance of the GFP would obscure the downregulation of Vsx2 mRNA. However, we found an excellent match between Vsx2 mRNA and Vsx2:GFP expression. We were surprised that sudden holes appeared in what was once rather uniform GFP labelling when Ath5 or Vsx1 turned on, as though GFP itself was suddenly being degraded. The same happens for Vsx2:dsRed. We have no explanation for this, but such findings might indicate that with a state change, such as occurs when a multipotent progenitor becomes lineage restricted, many nonessential proteins may be targeted for degradation or elimination. What causes the sudden downregulation of Vsx2 mRNA or loss of Vsx2:GFP is not known, but such state changes will certainly be an interesting topic for future investigations.

Pax6 has also been implicated as a multipotency factor for retinal progenitors as, like Vsx2, it is initially expressed in all early progenitors and only later becomes restricted to certain cell types [42]. Pax6 is essential for optic cup formation and so to investigate its role in RPC multipotency it was necessary to knock it out conditionally at late embryonic stages [5]. Results for such conditional knock-out mice revealed that without Pax6 all progenitors give rise to only a certain class of retinal cell, namely amacrine cells. How the absence of Pax6 reduces the competence of RPCs in this way or the mechanism by which Pax6 helps maintain the multipotent state is not clear, although from these results, a case can be made for a positive role for Pax6 in promoting multipotency. The fact that Vsx2 acts as a repressor for *vsx1 *[20], *mitf *[13], and photoreceptor-specific genes [12] led us to think that Vsx2 may repress a variety of transcription factors that affect fate restriction or competence states of retinal progenitors. We therefore expected to find that Vsx2 downregulation would be correlated with the expression of other lineage factors. To test this hypothesis *in vivo*, we crossed Tg(*vsx2:GFP*) with Tg(*ath5:RFP*) as Ath5 is expressed in a population of progenitors that divide once to generate RGCs and other cell types, including amacrine cells, horizontal cells and photoreceptors [30]. Our results show that Ath5 is expressed only in RPCs that profoundly downregulate Vsx2. *In vitro *assays show that Vsx2 binds to the *ath5 *promoter region, suggesting that Vsx2 directly represses *ath5*. Consistent with this, we found that Ath5 expression is upregulated in Vsx2 morphants. Interestingly, we also found that Foxn4 expression is upregulated in Vsx2 morphants. Foxn4 is a winged helix/forkhead transcription factor expressed in proliferative progenitors that specifically give rise to amacrine and horizontal cells in mice [33]. Foxn4 may thus also be a direct target of Vsx2 repression as suggested in Figure 15.

Zebrafish Vsx2 morphants have microphthalmia. This is consistent with the ocular retardation phenotype described previously in mice [8,9,25]. We did not study retinal proliferation in this paper, but key studies by other groups have shown that the effect of Vsx2 on cell proliferation is separable from its effect on cell fate [10,16]. When looked at in the context of the lineages studied here, Vsx2's effect on proliferation may be illuminating, for as committed progenitors downregulate Vsx2, they also downregulate cell cycle inhibitors, though probably indirectly. Thus, at the same time as they become restricted to particular lineages, progenitors lose proliferative potential. Cells in which Vsx2 stays on, however, retain proliferative potential, suggesting that Vsx2 cells may be more stem cell-like. Müller cells are the only cells that seem to maintain *vsx2 *expression at the level it is expressed in multipotent RPCs. Our results may thus explain why Müller cells can re-enter the cell cycle and produce various missing cell types in the damaged fish retina as described previously [43].

All cells types, including PKCβ1-positive cells and Vsx1 bipolar cells, are present in the retinas of Vsx2 morphants, although Vsx2:GFP (or Vsx2:Dsred) bipolars are reduced in number. In mice, there is a profound loss of bipolar cells in *ocular retardation *(*vsx2*) mutants. A possible explanation for this difference is that more bipolar cells in mice express Vsx2 than in fish [20]. Another, perhaps more likely possibility is that the morpholino gives an incomplete knock down of the Vsx2 protein, so the morphant phenotype is less severe than a mutant null phenotype. The importance of this result, however, may have more to do with the fact that Vsx2 is unnecessary for the differentiation of all other cell types, which fits with its proposed major role as an inhibitor of the specification of the progenitors of these cell types. In the absence of Vsx2, lineage determinants are derepressed, and so all cells, except perhaps those few bipolar cells that need to upregulate Vsx2, can continue to develop. lt would therefore be interesting to rescue cell proliferation in Vsx2 morphant fish, perhaps by knocking out p27 as in mice, and then ask whether the Vsx2:GFP bipolars that develop are of the S4 or S5 subtype.

If Vsx2 downregulation is required for the generation of committed progenitors, then it might be expected that misexpression of Vsx2 would interfere with the formation of all cell types except for bipolar cells and Müller cells. But testing such a prediction was complicated by the fact that Vsx2 is a potent repressor of its own transcription. Moreover, Vsx2 misexpression at early stages interfered with optic vesicle formation. In short-term explants of E17 mouse retina, virally induced misexpression of Vsx2 led to the overproduction of Müller cells and bipolar cells with a concomitant decrease in the number of photoreceptors and amacrine cells [3]. Livne-Bar *et al*. [16] transfected retinal cells in live mice at P0, examined their retinas at maturity and also showed an increase in bipolars, but this time more at the expense of rod cells. In both cases, the misexpression began during the period when most ganglion cells, amacrine cells, horizontal cells and cones have already been generated. In our zebrafish experiments, early RNA driven misexpression of Vsx2 in cells transplanted to wild-type hosts also resulted in an increase of bipolar cells at the expense of amacrine cells, whilst the heatshock driven misexpression of Vsx2 at later stages also increased the proportion of bipolar cells, but this time at the expense of photoreceptors. Such results are concordant with the mouse data, and suggest that the timing of overexpression is critical to the phenotype, as we might expect if Vsx2 acts like a gate-keeper on lineages that arise at different times in retinogenesis.

Beside its specific effect on bipolar cell fates, and keeping cells proliferative, Vsx2 represses transcription factors involved in specifying the progenitors of other lineages. This latter function, which is highlighted in this paper, suggests that Vsx2-positive RPCs may act like multipotent retinal progenitors that produce distinct Vsx2-negative progenitors, each with limited competence and proliferative potential.

## Materials and methods

### Animals

Zebrafish were maintained and bred at 26.5°C, and embryos were raised at 28.5°C and staged as previously described [44] in hpf. All embryos were treated with 0.003% phenylthiourea (Sigma, Gillingham, Dorset, UK) from 11 to 24 hpf to delay pigment formation in the eye.

Zebrafish transgenic lines Tg(*vsx2:GFP*) and Tg(vsx:dsRed) expressing GFP or dsRed under the promoter of *vsx2*, and a transgenic line expressing GFP under the control of *vsx1 *regulatory regions [26,28] were used in this study. Tg(*ath5:Gap-GFP) *and Tg(*ath5:Gap-RFP*) [31], expressing fluorescent proteins under the *ath5 *promoter, were also used. Double transgenic lines were created by crossing single transgenic lines to each other.

The zebrafish transgenic line Tg(*ptf1a*:*GFP*) expresses GFP under the control of the *ptf1a *promoter and was generated using BAC recombineering [45] and kindly provided to us by Professor Steven D Leach's laboratory (John Hopkins Medical Institutions, Baltimore, USA).

The use of zebrafish for this project was approved by the local ethical review committee at the University of Cambridge and was conducted under project licence PPL 80/2198, approved by the UK Home Office.

### Constructs

The *pCS2*+ vector (David Turner, University of Michigan) was used to subclone *vsx2 *IMAGE clone (ID number 5412931, IMAGE Consortium UK), using the primers 5'-GGG CTC GAG ATG ACA GGA AAG GAT GGG-3' and 5'-GGG CTC GAG CTA AGA GCT CTT TTC CTC-3'. The amplified fragments were digested with restriction enzyme *Xho*I and cloned in the *Xho*I site in the *pCS2 *vector. The cDNA of *vsx2 *was also subcloned in the *Xho*I site in the *pSGH2 *vector (obtained from Thomas Czerny, University of Veterinary Medicine, Vienna, Austria). A pCS2-*ath5*-*Myc *construct (gift from Ichiro Masai, RIKEN Institute, Japan) was used to obtain *ath5*-*Myc *RNA. For mosaic expression of fluorescent proteins and morphological characterization of single cells, embryos were injected with *vsx2:GFP *or *vsx1:GFP *BAC constructs and sometimes co-injected with *H2B-RFP *mRNA transcribed from a pCS2-*H2B-RFP *construct. DNA injections were made into the cell at the one-cell stage, whereas mRNA injections were made into the yolk at the one-cell to four-cell stage using a micromanipulator-mounted micropipette and a Picospritzer microinjector. *H2B:RFP *capped RNA was transcribed with SP6 RNA polymerase using the mMessage mMachine Kit (Ambion, Warrington, UK).

### Heat shock experiments

The *pSGH:vsx2 *(pSGH construct obtained from Thomas Czerny, University of Veterinary Medicine, Vienna) construct is a bi-directional meganuclease transgene vector containing a fragment with eight heat shock elements upstream of a CMV minimal promoter, driving the expression of *GFP *gene. In the opposite orientation it has a similar cassette with the same minimal promoter, untranslated regions and pA containing a polylinker where the cDNA of *vsx2 *was inserted. The heat shock elements have been described to provide heat stress inducibility, therefore driving basal expression of these genes [46,47]. The vector also included a meganuclease site. For the meganuclease system [48], DNA is co-injected with I-SceI meganuclease enzyme (0.5 unit/Al) in 1× I-SceI buffer (New England BioLabs, Hitchin, Hertfordshire, UK) [48]. Microinjection was performed into the cytoplasm of single cell stage embryos. After injections embryos were incubated at 28.5°C until the heat shock time. Heat shock was always done in 50 ml falcon tubes in the waterbath, with no more than 40 embryos per falcon. Embryos were heat shocked at 39°C for 1 hour at 28–29 hpf. GFP is detected approximately 2 hours after heat shock. As a control situation we used the empty vector only driving GFP.

### Morpholino injection

MOs were obtained from Open Biosystems (Huntsville, Alabama, USA). For the *vsx2 *gene (sequence accession number UA2898) the anti-*vsx2 *translation blocking MO (Vsx2aATGMO) used was MORPH0774 (PZF1245-8858272) with the sequence (5\'-3\') CCTTTCCTGTCATGGTGCCTCTGAG. Vsx2aSPMO1 and 2 are splicing blocker MOs, with sequences designed against the exon1/intron1 boundary. The sequence of SMO1 is (5\'-3\') ACATCATCTGAATCTGAGCTGGCAG and the sequence of SMO1 is (5\'-3\') AGGCATGTACCCCGTACCTGAGCTG. Binding should block the splicing of intron 1 and induce a premature stop codon in intron 1 after 35 extra codons (105 bp) and a stop codon in intron 2 after two codons (6 bp), resulting in a Vsx2 protein lacking in its main protein domains.

All MO oligonucleotides were reconstituted as 1 mM stock solutions in water. The ideal amount to be injected was determined analysing a range of concentrations between 0.8 and 4.8 ng, by yolk injection of 1–4 cell stage embryos [49]. Water injection was used as a negative control. The Vsx2 MOs showed concentration dependent effects and different degrees of phenotypes were achieved in different genetic backgrounds of injected fish with the Vsx2aATGMO, which could be normalized to equivalent phenotypes as judged by eye size by slightly adjusting the amount injected.

Injecting either of the two SMOs individually resulted in a low proportion of wrongly spliced proteins (checked by RT-PCR) and, expectedly, did not result in the same degree of phenotype as achieved by the translational blocking MO. Injecting higher amounts (limits of 16 ng for SMO1 and 8 ng for SMO2) was too toxic and resulted in the death of embryos. Co-injection of both SMOs prevented the majority of RNA from being spliced correctly (RT-PCR) and phenocopied the translational blocking MO.

### Bromodeoxyuridine labelling

Sustained bromodeoxyuridine (BrdU) labelling of transgenic embryos was performed to determine the birthdates of retinal bipolar cells. A minimum of 20 embryos were injected starting at 30, 42, 48, 54, 60 and 66 hpf, spanning the time period before the first bipolar cells are born to the end of retinal development. Anaesthetized embryos were injected with 3 nl 10 mM BrdU (Sigma) into the brain ventricle every 12 hours to maintain BrdU availability until 66 hpf. All embryos were fixed in 4% paraformaldehyde in 0.1 M PB (phosphate buffer pH 7.4) at 4°C overnight at 75 hpf. The total number of sections quantified ranged from 11 to 12 for Vsx1:GFP cells (total number of cells 1,209–1,864) and 17 to 25 for Vsx2:GFP cells (total number of cells 820–1,226).

### Immunohistochemistry

Whole-mount zebrafish embryos were fixed with 4% paraformaldehyde overnight at 4°C, rinsed, cryoprotected in 30% sucrose and embedded in OCT and cryosectioned at 10 or 14 μm thickness. All immunohistochemistry staining steps were performed at room temperature unless stated otherwise. For BrdU experiments, sections were treated with 100% methanol for 10 minutes and 2 N HCl for 10 minutes. Mouse anti-GFP antibody was used to recover the bleached GFP in the transgenic lines during this process.

For immunolabelling, sections were incubated in blocking buffer (10% heat-inactivated goat serum, 1% bovine serum albumin, 0.2% Triton X-100 in PBS) for 30 minutes at room temperature. Sections were incubated in primary and secondary antibodies were incubated for 2 hours at room temperature or overnight at 4°C. Antibody dilutions are described below. For whole-mount immunostaining, embryos were fixed overnight in 4% paraformaldehyde in PBS, and all subsequent washes were performed in PBS containing 0.2% Triton X-100. Further permeabilization was achieved by incubating the embryos in 0.25% trypsin-EDTA in Hanks balanced salt solution for 15–25 minutes at 0°C. Blocking and antibody dilution was as for sections. Antibodies were incubated for at least 36 hours at 4°C, with occasional shaking.

Primary antibodies used were diluted in blocking solution, as follows: mouse anti-Zn8 (anti-Ben/DM-GRASP, specific for RGCs in the differentiating neural retina; Zebrafish International Resource Center (ZIRC), Eugene, OR, USA; 1:100–1:500); mouse anti-Zn12 (University of Oregon; 1:200); mouse anti-zpr1 (University of Oregon; 1:200); mouse anti-zpr3 (University of Oregon, USA; 1:50); mouse anti-glutamine synthetase (Chemicon, Temecula, CA, USA; MAB302 1:50); rabbit anti-GFP (Molecular Probes, Eugene, OR, USA; 1:500); rabbit anti-PKCα (Santa Cruz Biotechnology sc-208, Heidelberg, Germany); rabbit anti-PKCβ1 (Santa Cruz Biotechnology sc-209 1:100); mouse Zrf1 (University of Oregon, USA; 1:5); rabbit anti-calretinin (Chemicon AB5054; 1:1,000); rat anti-BrdU (Abcam, Cambridge, UK; 1:50); rabbit anti-Sox2 (Chemicon AB5603; 1:200); mouse anti-5E11 (kind gift from Associate Professor James M Fadool, Florida State University, USA; 1:50); rabbit anti-Hermes (kind gift from Associate Professor Malgorzata Kloc, University of Texas MD, Anderson Cancer Center, USA; 1:400).

Secondary antibodies used were goat or donkey anti-mouse or anti-rabbit IgG conjugated to Cy3 (Chemicon) or conjugated to Alexa 488, 594 or 637 fluorophores (1:1,000–1:2,000 dilution; Molecular Probes). Nuclei were counterstained with 4',6-diamidino-2-phenylindole (DAPI). Sections were coverslipped with fluorosave mounting medium.

### Whole mount *in situ *hybridization

*In situ *RNA hybridization was performed as previously described [50]. *vsx1 *(IMAGE ID: 4145868) and *vsx2 *(IMAGE ID: 5412931) zebrafish genes IMAGE clones were obtained and the cDNAs subcloned independently into pCS2+ or pBluescript vectors, respectively. All new constructs were sequenced to confirm the correct gene sequence and digoxigenin-labelled riboprobes were subsequently synthesized from these constructs.

Hybridized probes were detected with nitroblue tetrazolium chloride/5-bromo-4-chloro-3-indolyl-phosphate, toluidine salt (NBT/BCIP) or Sigma Fast Red tablets. Whole-mount *in situ *hybridization was performed on Tg(*vsx2:GFP*) and Tg(*vsx1:GFP*) embryos. For this the probes were visualized with Fast Red followed by whole-mount antibody labelling for GFP (overnight incubation) visualized with anti-mouse IgG conjugated to Alexa 488.

### Blastomere transplantation

To follow single progenitors and their progeny we transplanted 10–50 blastomeres from labelled embryos (expressing *vsx2:GFP *transgene and/or injected with *H2B-RFP *mRNA to obtain a general nuclei labelling) into the animal poles of unlabeled blastulas. In brief, embryos were embedded in 2% methylcellulose on a coverslip, and cells were transferred from one donor to up to four hosts with a glass micropipette as described previously [51]. Embryos were incubated as usual, keeping the donor apart when necessary to identify the morphants by eye size phenotype morphologically.

### Imaging of fixed samples

Whole or partial embryo images were acquired on a dissecting stereo microscope equipped with epi-fluorescence (Leica MZ FLIII). Photomicrography of whole-mount eyes or sections was performed with either a laser confocal system (Leica TCS-NT confocal laser scanning microscope using a Leica 40×, 1.2 NA or Leica 63×, 1.2 NA water immersion objectives) or with Nikon fluorescence microscopes, equipped with cooled charge-coupled device (CCD) Hamamatsu Orca cameras and automated *z*-drive and fluorescence shutters. At the confocal microscope a 405 nm laser line was used to visualise DAPI, a 488 nm argon laser line was used to visualise GFP and Alexa 488 fluorophore, a 568 nm laser line was used for the Fast Red (*in situ *hybridisation), RFP or DsRed (transgenic lines) or Cy3 excitation, a 594 nm laser line was used for Alexa 594 fluorphore excitation and a 633 nm laser line was used to visualise Alexa 647 fluorophore. Emission was detected using individual de-scanned PMT detectors. Optical sections of 0.5 μm thickness were taken through a volume of the retina up to 100 μm in depth and Kallmann averaged four times. Image data were acquired and stored as TIFF files using Leica TCS NT or Leica LCS software. Brightness and contrast of images was adjusted using Adobe Photoshop.

### Eye phenotype measurements

The surface area of central retina sections of control and Vsx2 morphant at 72 and 96 hpf was measured in digital photographs taken in the same magnification using Image J 1.40 (Wayne Rasband, National Institute of Heath). Measurements of 20 central sections from different eyes for each condition were compared using a Student *t*-test.

### Cell counting and data analysis

Slides were examined using a Nikon Optiphot-2 microscope. Images were captured using confocal laser scanning microscope Leica SP2. Images were processed using Volocity (Improvision, Coventry, UK). Cells were categorized according to morphology and layer position. Ganglion cells must have the cell body in the GCL. Amacrine cells must have the cell body in the inner part of the INL and send processes to the (IPL) and not to the OPL. Bipolar cells should have their cell body in the outer part of the INL and send processes to the IPL and OPL. Horizontal cells must have their cell body in the outermost part of the INL, just under the OPL, and their nuclei normally are located in a horizontal position. Müller cells must have their cell body in the inner part of the INL but send processes spanning the whole retina. Photoreceptor cell bodies must be in the ONL.

Statistical analysis was carried out by quantifying the number of labelled cells in different retina sections, and summing the results for different cell types from each eye. A χ^2 ^test was carried out to test whether the distribution of cell types from one condition is different from another condition. A binominal distribution was also applied when there were significant differences in the 5% interval.

### Live imaging of whole-mounted embryos

Embryo processing and four-dimensional imaging were performed as described previously [30]. Usually, stacks about 40 μm thick, composed of optical sections separated by 1 μm, were taken every 5–10 minutes during an average period of 24–42 hours. Laser power was kept at a minimum (typically 9–20%) to avoid damaging the embryos. The four-dimensional data thus obtained were processed and analyzed with Volocity (Improvision, Coventry, UK). Unless stated otherwise in the figure legends, the images shown are maximum-intensity projections of the same confocal stack.

### RNA extraction and RT-PCR

RNA was isolated from zebrafish embryos in different stages of development. Until 72 hpf, RNA was extracted from whole bodies or only heads of groups of 10 embryos at the same stage of development. At 80 hpf, RNA was also extracted from whole bodies, heads and eyes removed from 10 embryos. Tissues dissected for RNA extraction were removed in modified Bart's saline 1×, MS222 (0.4 mg/ml) in silicone dishes. RNA was extracted using the RNeasy Micro Kit (QIAGEN, Crawley, West Sussex, UK). RNA was quantified using the Spectrophotometer NanoDrop 1000 (Thermo Fisher Scientific, Wilmington, Delaware, USA). Repetition of RT-PCRs with different samples resulted in the same trends.

The QIAGEN OneStep RT-PCR Kit was used to perform highly sensitive and specific RT-PCR reactions. RT-PCR was performed in a 25 μl reaction mixture using 50 ng of total RNA in each reaction. Primers used were: *crx *Fw-5'-CACCAAAACACGCTATCCAGACATATT-3' and Rv-5'-CTAGTGCCAGGGTCAGAGGTCAGAT-3'; *rx3 *Fw-5'-AGTCAATTCTGGGATTTAAGGGAGAGA-3' and Rv-5'-GCTGACCATTACAGTAGACCTACAAAC-3'; *foxn4 *Fw-5'-TTTAACCTGGAAGCATTAGGGACTCTC-3' and Rv-5'-AACGATAGGTTTTGTTCCTCCTTGTGT-3'; *bhlhb4 *Fw-5'-AAGGACAGACTATAACTTCGCCGATTC-3' and Rv-5'-CT TATCGTTGCAGTGTTTGAACAGGT-3'; *ath5 *Fw-5'-CCAGAGACCCGGAGAAGTTTGAGAGT-3' and Rv-5'-CCAGTCTTTCTGAGGATCCACGTGTC-3'; *vsx1 *Fw-5'-TCAGGGAACTCTCAAAAGAGGAAAAA-3' and Rv-5'-ACCTGTATCCTGTCCTCTGGTAGCTCT-3'; *vsx2 *Fw-5'-CCTGGAGGTATTCCGTCTTTCTACAGT-3' and Rv-5'-CTGGCT CAGTGAAGACTTTGACATTTT-3'; *EF1α *Fw-5'-CATACATCAAGAAGATCGGCTACAACC-3' and Rv-5'-GTAGTACCGCTAGCATTACCCTCCTTG-3'; *mitfa *Fw-5'-ACTTCAAATGACAAACACGATTCCAGT-3' and Rv-5'-GATAAGAGTCAAACTTGCCACATGGTC-3'. Optimal reaction conditions were: 1× 50°C 30 minutes, 1× 95°C 5 minutes, 30 cycles of 95°C 30 s, 55°C 30 s and 72°C 30 s, and a final extension of 10 minutes at 72°C.

### Electrophoretic mobility shift assay

*Vsx2 *and *luciferase *were translated *in vitro *using rabbit reticulocyte lysate (SP6 TNT Quick Coupled Transcription/Translation system, Promega, L2080, Southampton, UK). Double stranded probes were composed of two complementary 5'-Cy5-labelled single-stranded oligonucleotides that were annealed by combining 14 pmol of each single-stranded oligo in annealing buffer (20 mM Tris/HCl pH 7.6, 5 mM MgCl_2_, 1 mM DTT, 0.1 mM EDTA) and incubating 10 minutes at 70°C followed by 30 minutes at room temperature. Unlabeled competitor oligos were annealed accordingly. Probe sequences (sense strand) were: M2, 5'-cactttttaattaggtaatg-3'; M2mut, 5'-ccaaagttaattggaatgtc-3'; C2, 5'-ttttttttaattagcttact-3'. The labelled probe (0.07 pmol) was incubated with *in vitro *translated protein for 30 minutes at 24°C in a final volume of 20 μl (4 mM Tris/HCl pH 7.9, 80 mM KCl, 1 mM EDTA, 1 mM DTT, 12 ng/μl salmon sperm DNA (Stratagene, Stockport, Cheshire, UK), 2 μg poly(dI-dC) (Sigma). In competition experiments, 1 μg of additional poly(dI-dC) or a 100-fold excess of unlabeled competitor oligo (7 pmol) was added. Samples were mixed with 7 μl 50% glycerol and 10 μl/reaction were resolved on a native 4% polyacrylamide gel using 0.5× TBE running buffer. Gel was run at 150 V for 3.5 hours at 4°C. The Cy5 signal was scanned with a Typhoon 9400 fluoimager (Amersham Biosciences, Amersham, Bucks, UK) using a 670BP30 filter.

## Abbreviations

BAC: bacterial artificial chromosome; bHLH: basic helix-loop-helix; BrdU: bromodeoxyuridine; DAPI: 4',6-diamidino-2-phenylindole; EMSA: electrophoretic mobility shift assay; GCL: ganglion cell layer; GFP: green fluorescent protein; hpf: hours post-fertilization; INL: inner nuclear layer; IPL: inner plexiform layer; MO: morpholino; ONl: outer nuclear layer; OPL: outer plexiform layer; PB: phosphate buffer; PBS: phosphate-buffered saline; PKC: protein kinase C; RFP: red fluorescent protein; RGC: retinal ganglion cell; RPC: retinal progenitor cell; SMO: splicing MO.

## Competing interests

The authors declare that they have no competing interests.

## Authors' contributions

MV carried out the majority of the experiments, which will form the basis of her graduate dissertation, and wrote the first draft of the paper. PJ contributed to the *in situ *hybridisation experiments, did the work on classifying the bipolar cell types, birth-dating of Vsx1 and Vsx2 bipolar cells and contributed greatly to the figures and revisions of the text. DM did the work on the EMSAs. YK and SH made and did the initial characterisation of the Vsx2 and Vsx1 transgenic lines used in this study. WH supervised the work, which was done in his laboratory, and did the majority of the writing and revising of the manuscript.

## Supplementary Material

Additional File 1 **Development of Vx2:GFP in the retina of a Tg(*vsx2:GFP*) embryo**. In this movie, some cells downregulate GFP while a minority upreguate GFP strongly and begin to differentiate as bipolar cells. See also Figure 2. Click here for file

Additional File 2 **Vsx1:GFP; Vsx2:DsRed cells developing in wild-type hosts**. At the start of this movie Vsx2:DsRed has already been, or is in the process of being, downregulated in most Vsx1:GFP cells, although some faint DsRed can still be seen. Vsx1:GFP cells divide apically and then upregulate GFP and differentiate into groups of bipolar cells while, later, bright Vsx2:DsRed bipolars arise from Vsx1:GFP-negative progenitors to join the Vsx1:GFP cells. These cells likely arise from Vsx2:DsRed progenitors, but due to bleaching they cannot be followed until Vsx2:DsRed is upregulated in differentiating cells. See also Figure 5. Click here for file

Additional File 3 **Vsx2/Vsx1 down-/upregulation, followed by Vsx2 upregulation**. In this movie of a double transgenic Tg(*vsx1:GFP; vsx2:dsRed*) embryo, Vsx2:DsRed is initially expressed throughout the retinal neuroepithelium, but then Vsx1:GFP begins to be expressed in a population of cells while Vsx2:DsRed is simultaneously downregulated. Occasionally, it is possible to see GFP cells dividing at the apical surface. At around 55 hpf, a separate subpopulation of cells in the INL begins to upregulate Vsx2:DsRed. See also Figure 6. Click here for file

Additional File 4 **Time-lapse of Tg(*vsx2:GFP*) cells expressing H2B:RFP transplanted to a wild-type host**. Initially, all transplanted progenitor cells express Vsx2:GFP and H2B:RFP. Some of these cells downregulate Vsx2:GFP and then divide apically. The Vsx2-negative daughter cells of these divisions migrate and settle to the ONL, the inner INL and the GCL where they presumably differentiate as photoreceptors, amacrine cells and ganglion cells. Much later, some cells can be seen to upregulate Vsx2:GFP and differentiate into Vsx2-positive bipolar or Müller cells. See also Figure 8. Click here for file

Additional File 5 **Ath5:RFP progenitors downregulate Vsx2:GFP**. A Tg(*ath5:RFP;vsx2:GFP*) retina in which it is possible to see an Ath5:RFP progenitor that downregulates Vsx2:GFP and then divides once at the apical surface of the retina to produce one daughter that differentiates as an RGC. See also Figure 9. Click here for file
